# DABCO-Customized Nanoemulsions: Characterization, Cell Viability and Genotoxicity in Retinal Pigmented Epithelium and Microglia Cells

**DOI:** 10.3390/pharmaceutics13101652

**Published:** 2021-10-10

**Authors:** Ana R. Fernandes, Tiago dos Santos, Pedro L. Granja, Elena Sanchez-Lopez, Antonello Santini, Maria L. Garcia, Amelia M. Silva, Eliana B. Souto

**Affiliations:** 1 i3s—Institute for Research & Innovation in Health, University of Porto, R. Alfredo Allen 208, 4200-135 Porto, Portugal; anaritavfernandes@gmail.com (A.R.F.); tiago.f.santos@ineb.up.pt (T.d.S.); pgranja@i3s.up.pt (P.L.G.); 2 INEB—Biomedical Engineering Institute, University of Porto, Alfredo Allen 208, 4200-135 Porto, Portugal; 3 Faculty of Engineering, University of Porto, R. Dr. Roberto Frias, 4200-465 Porto, Portugal; 4 Centre for Research and Technology of Agro-Environmental and Biological Sciences, CITAB, UTAD, Quinta de Prados, 5001-801 Vila Real, Portugal; 5 Department of Pharmacy, Pharmaceutical Technology and Physical Chemistry, Faculty of Pharmacy, University of Barcelona, 08028 Barcelona, Spain; esanchezlopez@ub.edu (E.S.-L.); marisagarcia@ub.edu (M.L.G.); 6 Institute of Nanoscience and Nanotechnology (IN2UB), University of Barcelona, 08028 Barcelona, Spain; 7 Department of Pharmacy, Università di Napoli Federico II, Via D. Montesano 49, 80131 Napoli, Italy; asantini@unina.it; 8 Department of Biology and Environment, University of Trás-os-Montes e Alto Douro, UTAD, Quinta de Prados, 5001-801 Vila Real, Portugal; 9 CEB—Centre of Biological Engineering, Campus de Gualtar, University of Minho, 4710-057 Braga, Portugal

**Keywords:** cationic nanoemulsions, DABCO surfactants, quinuclidine surfactants, ocular administration, triamcinolone acetonide, age-related macular degeneration, HMC3 cell line, ARPE-19 cell line

## Abstract

Quaternary derivatives of 1,4-diazabicyclo[2.2.2]octane (DABCO) and of quinuclidine surfactants were used to develop oil-in-water nanoemulsions with the purpose of selecting the best long-term stable nanoemulsion for the ocular administration of triamcinolone acetonide (TA). The combination of the best physicochemical properties (i.e., mean droplet size, polydispersity index, zeta potential, osmolality, viscoelastic properties, surface tension) was considered, together with the cell viability assays in ARPE-19 and HMC3 cell lines. Surfactants with cationic properties have been used to tailor the nanoemulsions’ surface for site-specific delivery of drugs to the ocular structure for the delivery of TA. They are tailored for the eye because they have cationic properties that interact with the anionic surface of the eye.

## 1. Introduction

Nanoemulsions are a subcategory of emulsion characterized by a mean droplet size between 20 and 500 nm. Nanoemulsions are composed of two immiscible liquids in which the droplets of one liquid (dispersed phase or inner phase) are dispersed in another liquid (continuous phase or external phase) and can be classified into two types, namely, oil-in-water (o/w) and water-in-oil (w/o) [1]. In the production of emulsions and nanoemulsions, surfactants are usually required to stabilize the formulations and prolong their shelf life [2]. Surfactants are molecules that rest at the interface between oil and water to stabilize emulsions through different mechanisms, either by steric stabilization and/or by electrostatic repulsion [3]. Surfactants used to stabilize the nanoemulsions can be ionic or nonionic. The most commonly used ionic surfactants are sodium dodecyl sulfate (as anionic) and the cetyltrimethylammonium bromide (as cationic) and other typical nonionic surfactants, such as polysorbates and sorbitan esters. Other less common types of surfactants include natural biopolymers, such as whey protein and gum arabic [4,5]. Nanoemulsions can be obtained by high-energy methods or by low-energy methods. The high-energy methods require the use of high-pressure homogenization (HPH) and/or ultrasonication (US). In the HPH process, the size of the obtained droplets is governed by the pressure, design and size gab of the homogenization valve [6]. In the US process, the electrical input induces pressure fluctuations that produce cavitation bubbles. The break of the cavitation bubbles generates shear forces that promote the break of bigger drops into smaller drops [7]. In opposition to high-energy methods, low-energy methods only need the mixture of the components of the formulation using a stirrer [8,9].

Due to their properties, nanoemulsions are attractive delivery systems for poorly water-soluble drugs to improve their solubility and increase bioavailability [10,11]. In the nanoemulsions created to deliver hydrophobic or lipophilic drugs, the drug is dissolved in the oil phase to produce o/w nanoemulsions. Triamcinolone acetonide (TA) is a potent synthetic steroid with known anti-inflammatory and anti-angiogenic activities [12]. TA is approved by the FDA as a glucocorticosteroid, and has been widely used by intravitreal administration in the treatment of diabetic macular edema, vitreoretinopathy, uveitis, sympathetic ophthalmia, proliferative diabetic retinopathy and age-related macular degeneration (AMD) [13,14,15]. The intravitreal injection of TA is widely reported to reduce leakage from choroid neovascularization. Nevertheless, the hydrophobicity and lack of solubility of TA are a challenge for the development of ocular drug delivery systems. The increase in drug solubility will result in increased bioavailability, attributed to the enhanced permeation through ocular tissues and intracellular transport. Besides, the use of controlled/prolonged delivery systems may also contribute to reduce the systemic toxicity of the drug [16].

Age-related macular degeneration (AMD) is one of the main causes of severe and irreversible loss of vision in the elderly population [17]. In the last few years, efforts have been made to understand the pathophysiology of neovascularization in AMD. This knowledge allows developing new strategies in the progress of medical treatments for AMD. There are two types of AMD, namely, the nonexudative (dry) AMD and exudative (wet) AMD. Approximately 10–20% of patients with dry AMD progress to the wet form of AMD, in which pathologic choroidal neovascular membranes grow under the retina [18]. In wet AMD, there is a growth of abnormal new blood vessels from the choroidal vasculature. This specific process involves the presence of an angiogenic and inflammation stimulant inducing the wound-healing cascade [19]. Steroids inhibit the migration and the activity of the cells that stimulate the inflammatory response and wound repair. Another property of corticosteroids is the suppression of inflammation and the immune response, and they are able to reduce the permeability of the blood–retinal barrier (BRB). Additionally, some corticosteroids have an anti-angiogenic effect [20]. TA has been considered a safe and effective drug for ophthalmic administration, as TA is well tolerated by the ocular tissues and retains its activity for months after one intravitreal injection. Intravitreal injection is, however, linked to severe complications and side effects, such as subconjunctival hemorrhage, retinal detachments, vitreous hemorrhages, intraocular inflammation [21] and increased intraocular pressure [22], among others [23,24]. In this work, we describe the development of nanoemulsions to deliver TA into microglia and human retinal pigment epithelial cell line cells, with the aim to increase its therapeutic effect.

## 2. Materials and Methods

### 2.1. Materials

Polysorbate 80 (Tween 80^®^) was purchased from Uniqema (Everberg, Belgium). Soybean oil, glycerol and triamcinolone acetonide were purchased from Sigma-Aldrich (Steinheim, Germany). P188 (Kolliphor 188 was purchased from BASF Schweiz AG (Kaisten, Germany). Cationic surfactants (mono- and dicationic DABCO and quinuclidine) were produced at the Arbuzov Institute of Organic and Physical Chemistry of the FRC Kazan Scientific Center of Russian Academy of Sciences. Ultra-purified water was obtained from the Milli^®^ Q Plus system, home supplied. Human retinal pigment epithelial cell line (ARPE-19) and human microglial clone 3 cell line (HMC3) were obtained from the American Type Culture Collection (ATCC^®^) and used at passages 7–20 and 21–27, respectively. Dulbecco’s modified Eagle medium nutrient mixture F-12 (DMEM/F-12), fetal bovine serum (FBS), 100 U/mL penicillin, 100 μg/mL streptomycin, trypsin–EDTA and Hank’s balanced salt solution (HBSS) were purchased from Gibco (Invitrogen Corporation, Waltham, MA, USA). Amicon^®^ Ultra Centrifugal Filters, 0.5 mL 50 K, were purchased from Merck Millipore Ltd. (Darmstadt, Germany).

### 2.2. Production of Nanoemulsions

Nanoemulsions were produced based on the method described by Fernandes et al. [25]. Briefly, an oil phase composed of soybean oil, tween 80 and a DABCO surfactant was heated in a water bath at 50 °C, followed by its dispersion in an aqueous solution composed of glycerol, poloxamer 188 and water, heated in a water bath at the same temperature of 50 °C, using a probe sonication Qsonica 4435 Q55 Sonicator Microprobe, 1/4”, with 0.635 cm of tip diameter (Sonics vibracell, Newtown, Connecticut, CT, USA) for 5 min (Table 1). The final step was the transfer of the hot nanoemulsion to an ice bath (0 °C) to allow cooling down. For the production of TA-loaded nanoemulsions, the drug was added to the inner oily phase before dispersion into the aqueous phase.

### 2.3. Particle Size Parameters and Zeta Potential

The mean particle size and polydispersity index (PI) were determined in triplicate by dynamic light scattering (DLS) using the Zetasizer Nano ZS (Malvern, Worcestershire, UK) at 25 °C. The values are given as the mean of triplicate runs per sample. The nanoemulsions were diluted in Milli Q water to a suitable concentration (1:1) to prevent multiple scattering.

The zeta potential analysis (ZP) was taken by electrophoretic light scattering using a Zetasizer Nano ZS (Malvern, Worcestershire, UK). The samples were taken in a Flow Cell at 25 °C, diluted with Milli-Q water (1:1) to prevent multiple scattering. The software system incorporated the Helmholtz–Smoluchowski equation, which was used to calculate the ZP. The obtained values are presented as the mean of triplicate runs per sample.

### 2.4. Osmolality

The values of osmolality of the nanoemulsions were recorded using the EquipWescor Vapor Pressure Osmometer VAPRO equipment, model 5520 (Logan, Utah, UT, USA). Ten microliters of the nanoemulsion were positioned into a solute-free paper disc in the sample holder of the equipment. The cycle measurement initiated when the sample holder was pushed into the equipment, taking 80 s. The results of Vapro are displayed in standard international units, mOsm/kg.

### 2.5. Minimum Inhibitory Concentration

The in vitro antifungal activity of nanoemulsions was evaluated against *Candida albicans*, by estimating the minimum inhibitory concentration (MIC), as previously described by Pashirova et al. [26]. The antifungal assay was performed in Sabouraud dextrose broth (HiMedia Laboratories Pvt. Ltd. Mumbai, India) (density 2 × 10^4^ cfu/mL), composed of peptone (10 g) and glucose (40 g), dissolved in purified water (1 L). Broth was previously adjusted to pH 5.6, followed by autoclaving for 15 min at 121 °C. The experiments were carried out in triplicate.

### 2.6. Rheological Behavior

The rheology of nanoemulsions was analyzed by applying a frequency sweep test, using a Rheo Stress RS 100 (Haake Instruments, Karlsruhe, Germany), a cone-and-plate test geometry rheometer with a plate diameter of 20 mm and a cone angle 48°. An oscillation frequency sweep test was performed over a frequency range from 0 to 10 Hz. The storage modulus (G’), loss modulus (G’’) and the complex viscosity (η^*^) of nanoemulsions were established as a function of the frequency with a constant stress amplitude of 5 Pa, i.e., in the region of linear viscoelastic.

### 2.7. Encapsulation Efficiency and Loading Capacity

An indirect method was used to assess the encapsulation parameters of TA in the nanoemulsions. The latter were firstly submitted to ultracentrifugation, and the supernatant was measured in the plate reader to calculate the drug concentration. A calibration curve was made by diluting the TA in Milli-Q water with 20% of ethanol to ensure the total dilution (0.6–600 μg/mL). The calibration curve was built upon the readings of TA, at 240 nm, in a BioTek Synergy HT (BIOTek Instruments, Winooski, VT, USA) plate reader. The encapsulation efficiency (EE) and loading capacity (LC) of TA in nanoemulsions were calculated as follows:

(1) $$\mathrm{EE}\%=\frac{W_{TA}-W_{S}}{W_{TA}}\times 100$$

(2) $$\mathrm{LC}\%=\frac{W_{TA}-W_{S}}{W_{TA}-W_{S}+W_{O}}\times 100$$

where *W_TA_* is the mass of triamcinolone acetonide (TA) used to produce the loaded nanoemulsions, *W_O_* is the mass of soybean oil used for the production of nanoemulsions and *W_s_* is the mass of TA quantified in the supernatant. Briefly, nanoemulsions were firstly passed through centrifugal filter units with a cut-off of 50k, i.e., 50,000 nominal molecular weight limit (NMWL), and ultra-centrifuged for 15 min at 13,400× *g* in a Beckman Optima™ Ultracentrifuge (Optima™ XL, Indianapolis, IN, USA) and with quantification determined in the supernatant in the plate reader at 240 nm. Centrifugal filter units used in the ultracentrifugation had a cut-off of 50k, i.e., 50,000 nominal molecular weight limit (NMWL).

### 2.8. Surface Tension

For the determination of the surface tension, the optical contact angle was recorded using an OCA 15 plus (DataPhysics Instruments GmbH, Filderstadt, Germany). OCA 15 plus is a video-based optical contact angle measurement device provided with an electronic syringe and connecter to a video camera. SCA20 software (Data Physics Instruments GmbH, Germany) was used for image analysis and subsequent calculation of nanoemulsion surface tension.

### 2.9. Cell Culture

Human retinal pigment epithelial cell line (ARPE-19) and human microglial clone 3 cell line (HMC3) were used at passages 7–20 and 21–27, respectively. Both cell lines were grown in Dulbecco’s modified Eagle medium nutrient mixture F-12 (DMEM/F-12) supplemented with 10% fetal bovine serum (FBS), and 1% antibiotics (100 U/mL penicillin and 100 μg/mL streptomycin). The above-mentioned cell cultures were maintained in a humidified incubator at 37 °C with 5% CO_2_.

#### 2.9.1. Cytotoxicity Assay (AlamarBlue^®^)

ARPE-19 and HMC3 cells were plated at a density of 50,000 cells/well in a 96-well plate (Costar, Cambridge, MA, USA) and incubated at 37 °C for 24 h in culture medium. After this time, the cells were treated with 200 µL of medium containing different concentrations of TA or 200 µL of nanoemulsions at increasing concentrations (5%, 10% and 20%) for 24 h. Cells treated with fresh medium served as a negative control, and treatment with 1 mM H_2_O_2_, for 24 h, served as a positive control. Cell viability was assessed using AlamarBlue^®^ assay. Briefly, AlamarBlue^®^ is a cell viability assay with a blue reagent dye called resazurin. Resazurin is an indicator colorimetric that changes in response to a cellular metabolic reduction. Resorufin is the reduced form of resazurin and presents a pink color with a high fluorescent intensity that is proportional to the number of alive cells respiring. AlamarBlue^®^ is then a direct indicator of cell viability and consequently the cytotoxicity of the nanoemulsions. After 24 h incubation, the cells were treated with Alamar Blue solution (10%, *v*/*v*, in culture medium). After 4 h, absorbance was read at 530 and 590 nm using an absorbance microplate reader (BioTek Instruments, Winooski, VT, USA) and cell viability was determined as percent absorbance relative to untreated control cells (negative control).

#### 2.9.2. Comet Assay

ARPE-19 and HMC3 cells were plated at a density of 50,000 cells/well, and grown to confluence in 24-well plates for 7 days. Twelve hours before the experiment, the cells were treated with different nanoemulsions (at 20%). After the treatment with nanoemulsions, the isolation of cells was carried out, followed by the preparation of a single-cell suspension to embed the cells in agarose gel. After proceeding the lysis, the cells were submitted to alkaline treatment followed by horizontal electrophoresis (30 min, 35 volts, 300 mA), as described by Doktorovova et al. [27]. After neutralization, the samples were stained with Vista Green DNA Dye and visualized in Inverted Fluorescence Microscope/Coupling Stage, Zeiss Axiovert 200M (Carl Zeiss, Germany) using the 20× objective. The assay was performed using the OxiSelect^TM^ Comet Assay Kit (Cell Biolabs, Inc., San Diego, CA, USA). The results were analyzed using the OpenComet software (www.cometbio.org, accessed on 15 February 2021); the percentage of DNA in the tail and the olive tail moment (OTM) were used to determine the extent of DNA damage.

### 2.10. Statistical Analysis

The results are shown as the mean of three measurements ± standard deviation (S.D), whenever applicable. Two-way ANOVA followed by Tukey’s post hoc test was used for multi-group comparison. Student’s *t*-test was used for two-group comparisons. Statistical significance was set at *p* < 0.05. GraphPad Prism V9.0 InStat (GraphPad Software Inc., San Diego, CA, USA) was used to carry out the analysis.

## 3. Results and Discussion

Nanoemulsions are commonly referred to as an excellent alternative formulation to deliver lipophilic drugs to the eye to increase their bioavailability [28]. In this work, we formulated cationic nanoemulsions to provide a high encapsulation rate for the glucocorticoid TA, to enhance its stability, ocular penetration and bioavailability. In order to create an electrostatic interaction with the negatively charged cells of the ocular surface and then increase the residence time of TA in the locale of action, the surface charge of the nanoemulsion (defined by the zeta potential) should be positively charged. The interaction between cationic nanoemulsions with the cornea may offer the opportunity to create a depot that will control the release of the drug into deeper eye structures. Thus, the local residence time governs the drug passive diffusion [29]. Nine distinct cationic surfactants were used for the production of drug-free nanoemulsions. The composition of the developed formulations is given in Table 1 [30,31].

Pre-formulation studies were previously run, as described by Fernandes et al. [25], to obtain the optimal ratio of each ingredient (Table 1). In order to optimize the nanoemulsions, the concentration of each cationic surfactant was modified to their critical micelle concentration (CMC). Then, nine nanoemulsions were produced, each containing a different cationic surfactant.

The particle size and zeta potential of the produced nanoemulsions were monitored over a period of 120 days. The selection of the best formulation was based on the smaller size and lowest polydispersity index (PdI). The smaller the droplet size and PdI, the higher the nanoemulsion stability. The zeta potential (ZP) refers to the electrokinetic potential in colloidal systems and is determined as the electric potential in the interfacial layer of a dispersed medium versus the electrical potential of a stationary layer of the fluid attached to the dispersed particle. The pH of the medium, the ionic strength, the temperature and the concentration of any additives are the most important factors that affect ZP. ZP results can be correlated with the short and long shelf life and/or stability of the nanoemulsions. Emulsions that have high values, negative or positive, of ZP are electrically stable. Otherwise, emulsions with low ZP values are more prone to coagulate or flocculate, possibly leading to poor physical stability [32]. Typically, higher ZP values mean that repulsive forces surpass attractive forces, which result in stable formulations.

Table 2 depicts the size and zeta potential variation over time for the nine developed formulations. The mean droplet size of all developed nanoemulsions was within the range required for ocular administration, which should be between 150 and 300 nm [29]. After 120 days, stored at 4 °C, the PdI of all developed nanoemulsions was around 0.2 (data not shown), which ensures monodispersed formulations [33]. There were no significant differences between the mean particle size during the 120 days for all the formulations, S1–S9 (Table 2).

A zeta potential value between +20 and +40 mV is required to create an energy barrier between the droplets and to avoid coalescence, and these values are required in formulations to be used in ocular delivery [34]. The changes encountered in ZP in all developed nanoemulsions were attributed to the dynamic properties of cationic surfactants which detach/reattach from the surface of the droplets over time.

The mean size and zeta potential variations were also recorded for the nanoemulsions loading TA (TA-NE) (Table 3).

Osmolality was recorded for the TA-free and TA-loaded nanoemulsions (Table 4). A sample of each nanoemulsion was positioned in a solute-free paper disc in the sample holder of the equipment. The cycle measurement initiates when the sample holder is pushed into the equipment and takes 80 s. As described in the literature, the formulations for ophthalmic use should respect the limits of 171–1711 mOsm/kg to the osmolality [35]. However, some authors considered that values of osmolality lower than 100 or higher than 640 mOsm/kg could be considered irritants to the eye [36]. The osmolality of tears is between 280 and 293 mOsm/kg [37]. All the nanoemulsions with TA have osmolality values well tolerated by the eye, as opposed to some of the nanoemulsions without the drug in their composition.

The minimum inhibitory concentration (MIC) refers to the lowest concentration of an antimicrobial drug and/or formulation that is able to inhibit the growth of a microorganism after incubation. The species of *Candida* are the most common cause of invasive infections provoked by yeast. These species are the predominant causative pathogens of nosocomial bloodstream infections. In the case of ocular infections, the presence of *Candida* in ocular tissues causes a series of complications that potentially promote defects in the visual field. The antifungal treatment should thus start immediately [38]. *Candida albicans* is the most predominant species that causes infection in the eye, namely endogenous yeast endophthalmitis, for example [39], and about 10–25% of infections provoked by *Candida* result in ocular candidiasis [40]. The MIC values of the nanoemulsions against *Candida albicans* are shown in Table 5.

The lowest MIC (2.4 µg/mL) was recorded for F1, whereas the highest MIC (29.7 µg/mL) belonged to F5. Nevertheless, all the formulations had a low minimum inhibitory concentration, which indicates that all the nanoemulsions have a potential fungistatic effect.

**Figure 1:**
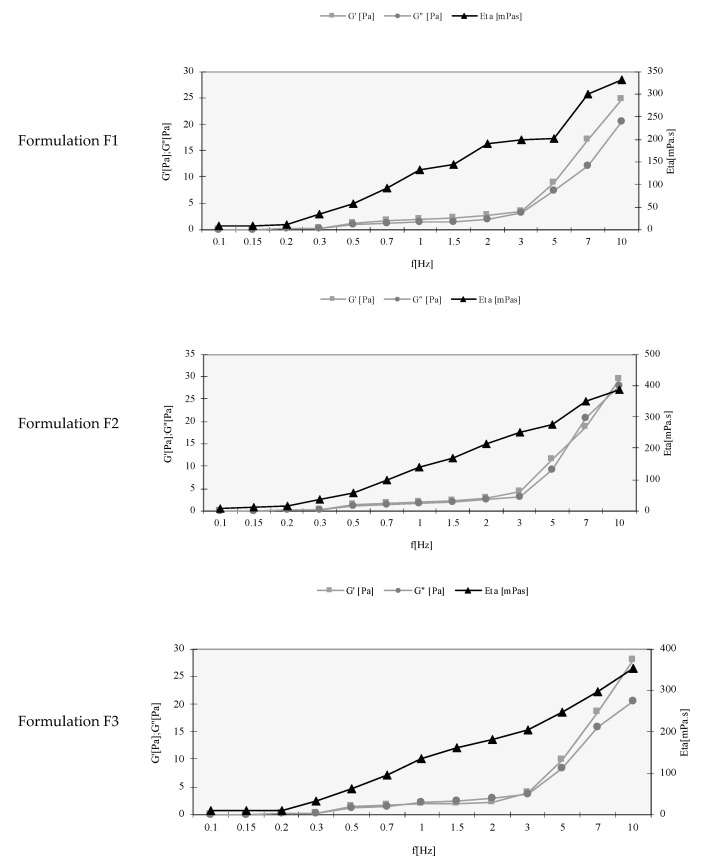

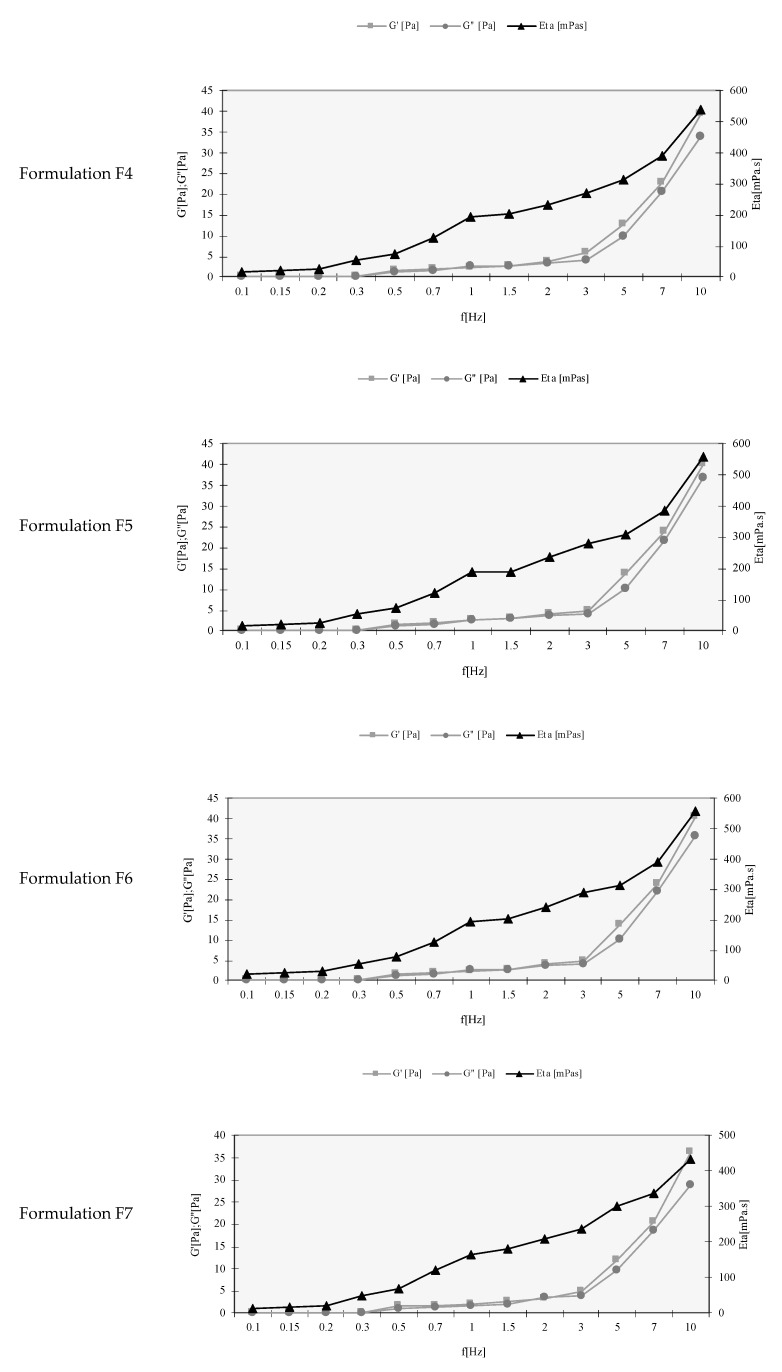

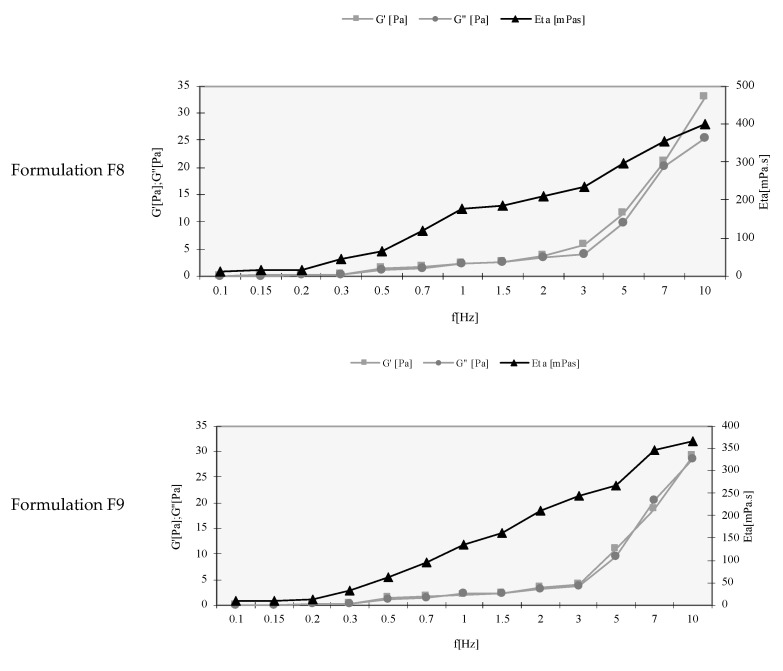
Rheological profiles of the storage modulus (G′), loss modulus (G″) and the complex viscosity (η^*^) of triamcinolone acetonide-loaded nanoemulsions at a constant stress amplitude of 5 Pa.

Considering that all TA-loaded nanoemulsions have antifungal properties, they were subject to rheological analyses. A frequency sweep test is a useful tool as it allows the determination of the viscoelastic properties of a sample as a function of timescale. The parameters obtained are described as storage elastic modulus (G′), the viscous loss modulus (G″) and the complex viscosity (η* or ETA). The G′ is normally used as a measure of the elastic component of the sample, and the loss modulus is used as a measure of the viscous component [41]. The frequency sweep test was conducted at a frequency range of 0.1 to 10 Hz and a constant stress amplitude of 5 Pa. The frequency range was the limit of the linear viscoelastic region determined before the beginning of the assay. The linear viscoelastic region is the range in which the assay can be carried out without destroying the sample, i.e., its structure. The results for each nanoemulsion are shown in Figure 1.

The behavior is similar in all the developed nanoemulsions containing the glucocorticoid. The profiles show that the elastic modulus (G′) is dominant over the viscous modulus (G″) in all the samples and is dependent on the frequency applied. The storage and loss modulus illustrated an increasing trend as a consequence of increasing the frequency. At low frequencies, all nanoemulsions present a low modulus that increases at high frequencies. The increase in the frequency was followed by an increase in viscosity in all the samples studied. It is described that at higher frequencies, the time to enable polymer chains to untangle is limited and their mobility is compromised, whereas at low frequencies they have the time necessary to untangle and are more mobile. Therefore, the higher frequencies are responsible for the increase in elasticity once the polymer chains have less mobility, and consequently are incapable of untangling to initiate the deformation of the sample [42]. The rheological results show that at the maximum frequency studied, i.e., 10 Hz, the value of G″ is smaller than G′, and, as described previously, both are dependent on the frequency, which indicates short relaxation times [43]. These relaxation times are a characteristic typical of viscoelastic samples.

The next step was the determination of the encapsulation parameters for all nine formulations with viscoelastic properties. The results are summarized in Table 6.

The initial concentration of TA was 50 µg/mL. The lowest %EE and %LC were obtained for F2, whereas the highest values were recorded for F9. The high encapsulation parameters recorded for all nanoemulsions were attributed to the lipophilic character of the drug, which was kept within the inner oil phase of nanoemulsions during their production.

**Figure 2:**
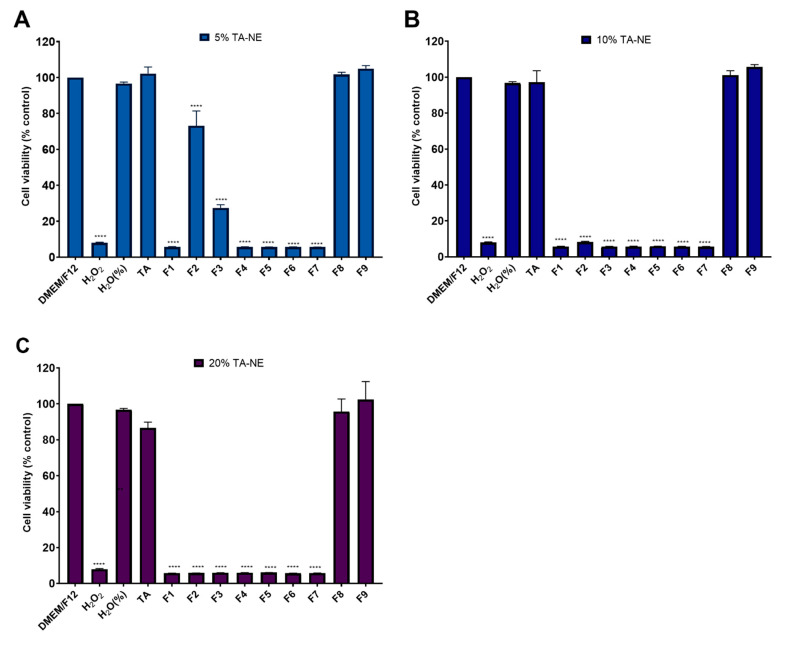
ARPE-19 cell viability upon exposure to TA-loaded nanoemulsons (F1–F9) recorded after 24 h of incubation. Culture media DMEM/F12 and 1 mM H_2_O_2_ were used, respectively, as negative and positive controls. The other control was performed by adding water to the culture medium, at the same percentage as that present in the nanoemulsions (%H_2_O), i.e., 5% (**A**), 10% (**B**) and 20% (**C**). The results are the mean of three replicates, and the bars represent the the mean ± standard deviation (S.D.). Statistical significance; **** *p* < 0.0001 vs. negative control (DMEM/F12). H_2_O_2_: hydrogen peroxide; TA-NE: nanoemulsions loading TA.

**Figure 3:**
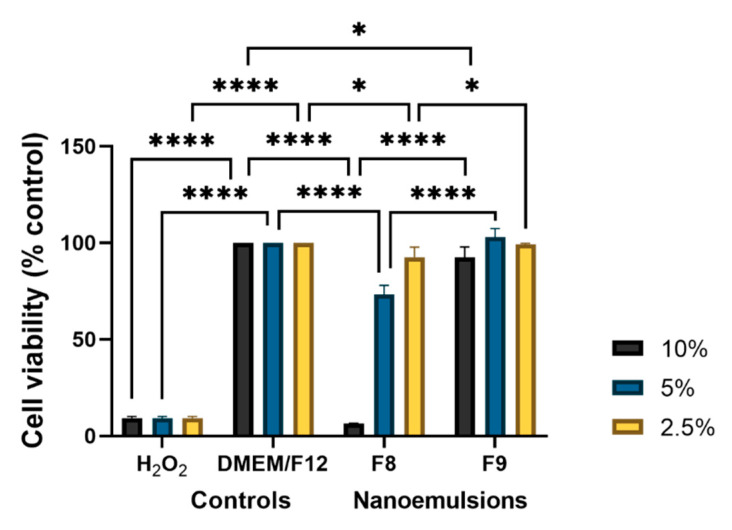
HMC3 cells viability after exposure to F8 and F9 for 24 h, at three different concentrations (10%, 5% and 2.5%). Cell culture medium (DMEM/F12) and culture medium supplementation with 1 mM H_2_O_2_ were used, respectively, as negative and positive controls. The results are the mean of three replicates, and the bars represent the mean ± standard deviation (SD). Statistical significance * *p* < 0.05; **** *p* < 0.0001. Abbreviation: H_2_O_2_, hydrogen peroxide.

Aiming at the evaluation of the potential cytotoxic effect of TA-NE, the ARPE-19 and HMC3 cell lines were exposed to the samples for 24 h. The effect of the nanoemulsions on the viability of the cells was measured by AlamarBlue^®^ assay. In vitro cytotoxicity results are depicted in Figure 2 and Figure 3 for ARPE-19 and HMC3 cell lines, respectively.

The hydrogen peroxide (H_2_O_2_) was used as a positive control (at 1 mM, in culture medium, for 24 h) and culture media, DMEM/F12, as a negative control. The other control was performed by adding water to culture medium, at the same percentage as that present in the nanoemulsions, i.e., 5%, 10% and 20%. After the administration in the eye, the nanoemulsion suffers a physiological dilution process. The dilution that occurs is 1:5 (*v*/*v*) [44], i.e., at 20%. Thus, the study was performed with nanoemulsions at 20% and two additional dilutions, 10% and 5%. The results show that the nanoemulsions produced with the surfactants 8 and 9, i.e., F8 and F9, respectively, were non-toxic for the ARPE-19 cell line at all tested concentrations (Figure 2). These results are in line with the concentration of TA used in all the dilutions, as the viability of the cells exposed to free TA was not compromised (Figure 2). Thus, the amount of TA used in the production of the nanoemulsions was considered to be safe with no toxic effect on ARPE-19 cells.

After obtaining the cytotoxicity results for the ARPE-19 cells, the cytotoxic effect of F8 and F9 was studied in the HMC3 cell line (Figure 3). As the drug needs to reach microglial cells, the tested nanoemulsion concentrations in HMC3 cells were 10%, 5% and 2.5%. It is expected that the drugs administrated to the eye suffer dilutions with the lachrymal fluids. Again, the cells were seeded in a 24-well plate for 7 days, with replacement of the medium every two days. After 7 days, the medium was changed, and the cells were exposed to nanoemulsions 8 and 9 (F8 and F9) for 24 h. After this period of incubation, the resazurin (10%) was added to each well, for 4 h, and then the plate was read at 530 and 590 nm using a fluorescence microplate reader.

Figure 3 shows that the higher the dilution, the higher the cytotoxicity of nanoemulsions, i.e., there is an increase in the cell viability with the decrease in concentration. With respect to the results obtained for 10% F8 and 10% F9, i.e., the more concentrated ones, compared to the results obtained in the cytotoxicity assay for the ARPE-19 cell line, the viability of the HMC3 cells is much more compromised in the case of F8, and there was a decreased viability in the cells exposed to F9. For the other concentrations (5% and 2.5%), there was an improvement in the viability of the cells compared to the most concentrated nanoemulsion. However, in the case of 5%, the viability of the HMC3 cell was lower than that obtained for APRE-19 cells in the same conditions. These nanoemulsions are thus more harmful to the microglial cells (HMC3) than to the retinal pigment epithelium cells (ARPE-19).

The surface of the eyes is covered with tear film. The tear film is a thin fluid layer that is the interface of the eye surface with the environment, and is approximately 3 μm thick and 3 μL in volume [45]. The tear film is constituted of three layers, a lipid layer, water layer and mucin layer. These constituents keep the eye healthy and without infections. In terms of surface tension, the tear film is normally destabilized when the value of the surface tension of the eyedrops used is lower than the value of the tear film. The surface tension could be described as the ability of a surface of a portion of liquid to be attracted by another surface or portion of liquid. Higher surface tension makes stronger interactions between the molecules of the liquids. The temperature decreases the surface tension due to the interactions inside the liquid being lower when compared to the heat-moving forces which promote the stability of the formulations [25].

The surface tension of the lachrymal fluid is around 40 to 50 mN/m [44]. Surface tension measurements were carried out at 36.7 °C with n = 14 for both nanoemulsions. The surface tension of the prepared TA nanoemulsions are 41.05 ± 2.06 mN/m for F8 and 43.37 ± 1.46 mN/M for F9. Lallemand et al. [29] reported that all the cationic nanoemulsions for ocular delivery should have surface tension similar to the tears, which confirms the potential of the produced nanoemulsions.

**Figure 4:**
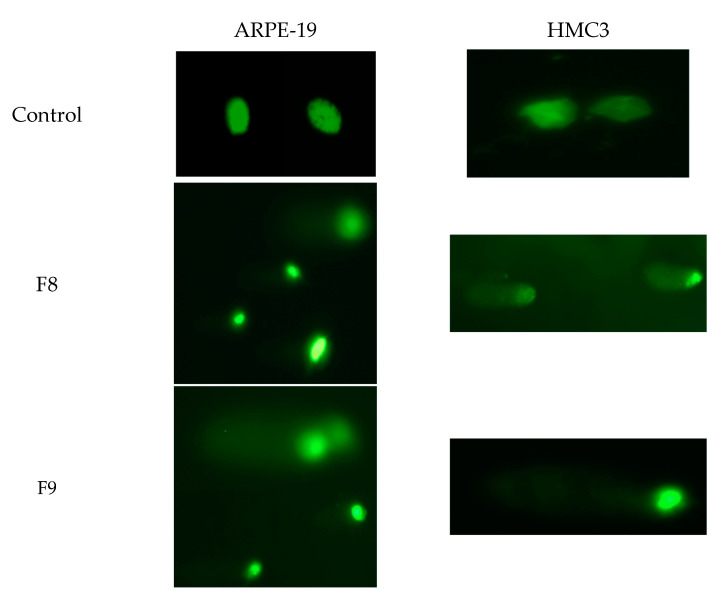
Genotoxic effects induced by nanoemulsions with TA in ARPE-19 and HMC3 cell lines. Images obtained with 20× objective.

**Figure 5:**
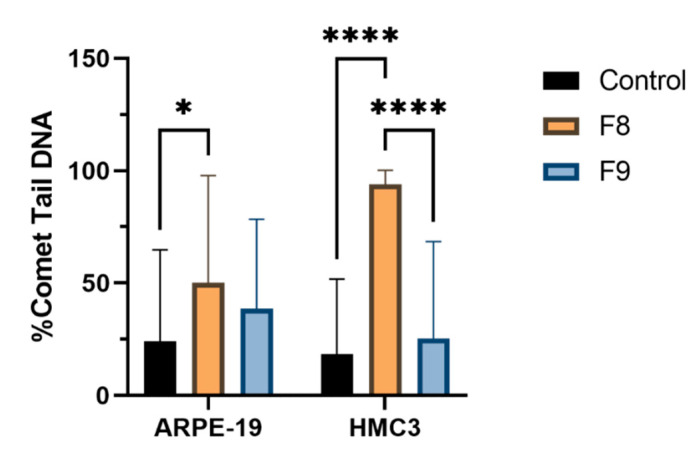
Genotoxic effects induced by F8 and F9 nanoemulsions in ARPE-19 and HMC3 cell lines. The bars indicate the % of DNA in the comet tail (mean ± SD). Mean was calculated by the OpenComet software. Statistical significance: * *p* < 0.05; **** *p* < 0.0001.

The comet assay reveals the genotoxic effects of nanoemulsions with TA on the ARPE-19 and HMC3 cell lines. The alkaline comet assay is a sensitive assay to detect DNA damage and has the ability to sensitively measure the strand breaks and other lesions that are converted into strand breaks under alkaline conditions. The comet assay does not directly measure the number of specific DNA lesions but rather measures the migration of DNA in the agarose gels as a result of the relaxation induced by strand breaks under alkaline conditions. The results shown in Figure 4, obtained with cells exposed to nanoemulsions at 20%, illustrate the comet tails indicative of DNA damage. In order to quantify the extent of induced DNA breaks in the cell lines, the intensity of the comet tails was scored (Figure 5).

As shown in Figure 5, in both cell lines, nanoemulsions at 20% increase the % of DNA in the comet tail as compared to the respective control. The scoring of comet tails in terms of % DNA in the tail showed a significant increase in DNA damage, after exposure to 20% F8, of HMC3 cells. In the case of F9, there was no significant DNA damage when compared to the control in both cell lines; thus, F9 does not lead to genotoxic effects. If we related the cytotoxicity and genotoxic assays, the nanoemulsion F8 presented higher toxicity in both cell lines (Figure 2 and Figure 3); hence, the increase in genotoxicity in the comet assay with F8 corroborates the previous results. The distance between the center of the head and the center of the tail, i.e., the olive tail moment, is represented in Table 7. The olive tail moment is defined as the product of the tail length and the fraction of total DNA present in the tail. The tail moment includes an amount of the lowest detectable size of migrating DNA and the quantity of broken pieces of DNA. The quantity of migrating DNA is revealed by the comet tail length and the amount of broken pieces of DNA is exposed by the intensity of DNA in the tail of the comet [46]. The increase in the olive tail moment represents higher DNA damage. In both cell lines, i.e., ARPE-19 and HMC3, F8 caused increased DNA damage.

## 4. Conclusions

The residence time of a cationic nanoemulsion is prolonged on the ocular surface, attributed to the electrostatic attraction between the cationic lipid nanodroplets and the negatively charged ocular surface. These typical properties of the produced nanoemulsions are beneficial for the administration of drugs on the ocular surface. In this work, we successfully developed a suitable nanoemulsion for the ocular delivery of triamcinolone acetonide. Both nanoemulsions, F8 and F9, were found to be stable for 2 months when stored at 4 °C, and have osmolality values compatible with ocular administration. The surface tension was 41.05 ± 2.06 mN/m for F8 and 43.37 ± 1.46 mN/M for F9, which are similar to the values of the lachrymal fluid. The in vitro results of the cytotoxic assay show high values of viability for ARPE-19 and HMC3 cells, after administration of F8 and F9 in different concentrations, which translates the safety of the nanoemulsions produced. However, in the comet assay (genotoxic test), the F8 revealed higher DNA damage in both cell lines (ARPE-19 and HMC3) when compared to the results obtained for F9 in the same concentration. Cationic nanoemulsions have been described as one of the most suitable formulations for ocular drug delivery to target both the anterior and posterior segments of the eye, attributed to their interaction with the corneal membranes, increasing the retention time of drugs in the eye. Aiming to avoid the risk of toxicity commonly reported with the use of cationic surfactants in drug formulations, in this work we developed nanoemulsions based on the use of the surfactant at its critical micelle concentration (CMC), i.e., the concentration at which micelles are spontaneously formed. A higher CMC of the cationic surfactant (S8) was used for F8 when compared to F9, which may be responsible for the increased genotoxicity reported for F8. From our studies, we select F9 as the most suitable cationic nanoemulsion for ophthalmic administration due to its nontoxic profile in the resazurin assay and its lower genotoxicity in the comet assay.

## Figures and Tables

**Table 1:** Composition of the developed nanoemulsions.

Ingredient	Nanoemulsions								
	S1	S2	S3	S4	S5	S6	S7	S8	S9
CMC (mM)	1.00	0.33	0.8	2.00	3.00	11.00	4.00	0.12	0.08
SO (% m/V)	2.00	2.00	2.00	2.00	2.00	2.00	2.00	2.00	2.00
T80 (% m/V)	0.20	0.20	0.20	0.20	0.20	0.20	0.20	0.20	0.20
P188 (% m/V)	0.01	0.01	0.01	0.01	0.01	0.01	0.01	0.01	0.01
Glycerol (% m/V)	1.50	1.50	1.50	1.50	1.50	1.50	1.50	1.50	1.50

Caption: S1–S9, DABCO and quinuclidine surfactants (for chemical structure, please refer to Fernandes et al. (2021) [25]); CMC, critical micellar concentration; SO, soybean oil; T80, Tween 80; P188, Poloxamer 188).

**Table 2:** Mean size and zeta potential variations of the developed nine nanoemulsions without TA, stored at 4 °C, over a period of 120 days. Results are the mean of three replicates ± standard deviation (S.D.). Statistical significance * *p* < 0.05; ** *p* < 0.01; *** *p* < 0.001; **** *p* < 0.0001 vs. day 0.

Mean Particle Size (nm)									
Day	S1	S2	S3	S4	S5	S6	S7	S8	S9
**0**	220.7 ± 2.9	242.3 ± 8.5	257.3 ± 8.4	245.3 ± 7.7	226.9 ± 4.4	243.3 ± 4.7	233.0 ± 4.7	251.6 ± 9.6	241.2 ± 3.6
**1**	231.4 ± 4.8	241.4 ± 3.9	263.7 ± 7.9	242.4 ± 8.8	231.1 ± 3.9	240.4 ± 8.0	230.4 ± 7.9	257.8 ± 7.9	252.3 ± 3.4
**3**	224.6 ± 4.3	240.8 ± 5.9	265.6 ± 9.7	242.9 ± 5.6	235.3 ± 9.0	239.3 ± 6.9	236.9 ± 8.7	254.5 ± 6.6	251.1 ± 5.8
**7**	234.7 ± 9.1	241.5 ± 5.5	263.6 ± 7.4	245.9 ± 7.6	233.4 ± 6.7	242.0 ± 6.9	234.7 ± 12.1	258.0 ± 0.9	253.2 ± 6.4
**14**	231.9 ± 9.0	242.2 ± 6.6	255.2 ± 9.8	244.7 ± 11.2	233.5 ± 7.2	240.8 ± 8.5	229.8 ± 9.2	256.9 ± 8.1	250.9 ± 6.8
**28**	227.4 ± 10.5	241.1 ± 4.0	265.6 ± 8.5	246.5 ± 9.4	231.2 ± 6.0	239.0 ± 3.5	230.8 ± 5.4	255.3 ± 7.9	253.2 ± 3.8
**60**	231.6 ± 9.6	240.1 ± 7.9	264.6 ± 6.2	246.0 ± 5.7	230.9 ± 8.6	230.2 ± 5.6	235.2 ± 5.9	254.3 ± 5.2	245.3 ± 1.3
**120**	215.4 ± 1.0	232.2 ± 3.5	257.0 ± 7.2	235.1 ± 8.3	226.7 ± 3.5	233.3 ± 7.1	223.7 ± 7.9	236.3 ± 5.4	243.6 ± 6.1
**ZP (mV)**									
**Day**	**S1**	**S2**	**S3**	**S4**	**S5**	**S6**	**S7**	**S8**	**S9**
**0**	71.6 ± 0.4	51.1 ± 3.1	41.0 ± 1.5	49.5 ± 0.7	63.8 ± 1.5	41.0 ± 1.5	68.4 ± 1.5	39.9 ± 1.2	44.3 ± 0.5
**1**	53.8 ± 0.1 ****	44.3 ± 2.0 ****	35.6 ± 0.7 **	46.3 ± 1.3	60.8 ± 2.1	43.7 ± 1.2	68.4 ± 2.1	35.5 ± 1.3 *	42.2 ± 1.2
**3**	64.2 ± 2.7 ****	43.4 ± 1.7 ****	31.4 ± 1.7 ****	48.1 ± 0.7	59.6 ± 2.1	45.2 ± 1.8	64.9 ± 0.9	23.7 ± 0.9 ****	34.1 ± 1.0 ****
**7**	53.4 ± 2.6 ****	37.8 ± 0.4 ****	29.9 ± 0.8 ****	47.7 ± 1.3	57.9 ± 2.6 **	38.6 ± 1.9	62.2 ± 0.7 ***	28.9 ± 2.1 ****	30.5 ± 0.2 ****
**14**	62.8 ± 1.3 ****	45.6 ± 1.6 **	37.1 ± 1.9	47.5 ± 0.8	58.7 ± 2.0 **	38.9 ± 0.4	69.6 ± 1.3	29.7 ± 2.4 ****	26.2 ± 0.9 ****
**28**	60.1 ± 1.0 ****	38.9 ± 0.9 ****	29.4 ± 0.6 ****	44.7 ± 1.6 *	59.4 ± 2.2 *	36.9 ± 1.3	66.5 ± 0.7	31.1 ± 0.7 ****	24.3 ± 0.8 ****
**60**	56.1 ± 0.9 ****	34.2 ± 1.1 ****	27.4 ± 1.6 ****	39.1 ± 2.3 ****	53.9 ± 1.9 ****	40.8 ± 0.3	59.3 ± 1.8 ****	33.8 ± 0.6 ***	29.5 ± 1.3 ****
**120**	69.3 ± 3.3	52.7 ± 0.9	43.1 ± 0.8	58.0 ± 2.1 ****	58.6 ± 3.6 **	35.7 ± 0.3 **	67.0 ± 0.7	36.7 ± 0.45	22.9 ± 0.9 ****

**Table 3:** Mean size and zeta potential variations of the developed nine nanoemulsions with TA, stored at 4 °C over a period of 120 days. Results are the mean of three replicates ± standard deviation (S.D.). Statistical significance * *p* < 0.05; ** *p* < 0.01; *** *p* < 0.001; **** *p* < 0.0001 vs. day 0.

Mean Particle Size (nm)									
Day	F1	F2	F3	F4	F5	F6	F7	F8	F9
**0**	224.1 ± 1.3	234.7 ± 7.9	257.9 ± 7.2	205.0 ± 5.6	223.0 ± 6.9	247.6 ± 6.8	227.7 ± 5.6	232.6 ± 3.5	218.3 ± 2.5
**1**	233.5 ± 7.3	230.8 ± 4.7	251.9 ± 9.5	204.1 ± 4.3	223.1 ± 7.9	253.0 ± 11.1	224.8 ± 6.5	242.3 ± 4.3	217.4 ± 4.7
**3**	230.9 ± 7.4	233.6 ± 9.5	259.0 ± 8.3	207.9 ± 5.2	223.2 ± 7.5	254.7 ± 10.0	223.1 ± 6.5	246.8 ± 5.9	215.5 ± 6.8
**7**	233.7 ± 2.6	231.5 ± 6.9	260.5 ± 7.0	209.9 ± 6.2	221.2 ± 5.8	250.8 ± 5.6	227.6 ± 5.6	245.3 ± 5.4	218.7 ± 5.5
**14**	229.4 ± 7.5	233.1 ± 5.5	254.7 ± 4.6	205.4 ± 3.6	221.0 ± 6.6	249.4 ± 6.3	225.5 ± 5.2	229.7 ± 4.3	216.9 ± 2.7
**28**	241.0 ± 11.2 *	230.0 ± 6.0	254.1 ± 6.6	206.5 ± 6.4	219.9 ± 5.0	248.3 ± 3.9	216.1 ± 5.2	242.9 ± 5.7	219.4 ± 5.6
**60**	226.9 ± 3.2	234.3 ± 6.6	258.5 ± 9.1	209.7 ± 3.7	220.4 ± 6.0	243.3 ± 7.7	221.6 ± 6.8	243.3 ± 6.6	220.0 ± 5.8
**120**	227.4 ± 6.7	223.3 ± 4.9	243.6 ± 3.8	202.8 ± 3.3	210.1 ± 6.3	243.3 ± 1.2	214.7 ± 5.3	230.5 ± 1.7	213.0 ± 5.3
**ZP (mV)**									
**Day**	F1	F2	F3	F4	F5	F6	F7	F8	F9
**0**	68.2 ± 1.9	41.2 ± 0.4	44.6 ± 1.2	65.1 ± 1.2	65.1 ± 2.7	48.3 ± 1.1	55.9 ± 1.4	44.9 ± 0.5	41.5 ± 0.2
**1**	46.6 ± 1.1 ****	50.3 ± 1.9 ****	56.8 ± 0.4 ****	66.4 ± 1.7	62.4 ± 2.3	44.2 ± 0.6 *	56.8 ± 1.5	41.3 ± 2.9	42.5 ± 0.5
**3**	58.6 ± 1.7 ****	43.0 ± 1.0	45.2 ± 1.0	59.5 ± 1.7 ***	60.2 ± 2.3 **	43.9 ± 0.8 *	61.9 ± 1.0 ***	32.0 ± 1.0 ****	40.8 ± 0.9
**7**	51.4 ± 1.8 ****	36.8 ± 1.3 *	39.7 ± 0.1 **	59.1 ± 2.1 ***	57.8 ± 4.2 ****	43.7 ± 0.5 **	55.6 ± 1.1	31.6 ± 0.9 ****	41.0 ± 1.7
**14**	60.2 ± 2.9 ****	37.5 ± 1.4	43.9 ± 2.0	61.9 ± 0.9	57.8 ± 2.1 ****	40.6 ± 1.2 ****	53.0 ± 1.4	46.1 ± 0.5	40.0 ± 0.9
**28**	36.5 ± 1.1 ****	36.6 ± 0.6 **	51.2 ± 1.7 ****	60.5 ± 1.2 **	57.6 ± 1.7 ****	41.9 ± 1.3 ****	71.2 ± 2.2 ****	37.0 ± 1.5 ****	35.8 ± 0.5 ***
**60**	52.6 ± 1.8 ****	36.4 ± 1.3 **	48.8 ± 1.0 *	49.5 ± 1.6 ****	56.5 ± 2.2 ****	43.5 ± 1.0 **	54.5 ± 0.4	32.6 ± 0.7 ****	32.5 ± 0.4 ****
**120**	61.1 ± 2.0 ****	48.6 ± 1.7 ****	58.5 ± 1.1 ****	63.9 ± 1.8	66.5 ± 1.3	38.9 ± 1.8 ****	64.2 ± 1.1 ****	35.7 ± 0.4 ****	31.7 ± 0.8 ****

**Table 4:** Osmolality of nanoemulsions with and without TA.

Nanoemulsion without TA	mOsm/kg	Nanoemulsion with TA	mOsm/kg
S1	140	F1	186
S2	160	F2	176
S3	147	F3	186
S4	164	F4	172
S5	172	F5	180
S6	190	F6	188
S7	183	F7	170
S8	160	F8	184
S9	153	F9	189

**Table 5:** Minimum inhibitory concentration (MIC) of triamcinolone acetonide-loaded nanoemulsions determined against *Candida albicans*.

TA-Nanoemulsions	Fungistatic Activity (MIC), µg/mL
F1	2.4 ± 0.1
F2	11.9 ± 1.3
F3	6.5 ± 0.5
F4	2.1 ± 0.1
F5	29.7 ± 1.7
F6	4.8 ± 0.7
F7	6.3 ± 0.1
F8	8.1 ± 0.2
F9	9.8 ± 1.1

**Table 6:** Encapsulation efficiency and loading capacity of the TA into nanoemulsions.

	F1	F2	F3	F4	F5	F6	F7	F8	F9
% EE	95.438 ± 0.070	83.650 ± 6.874	91.494 ± 0.989	91.083 ± 4.501	93.000 ± 0.675	86.416 ± 1.422	93.850 ± 1.233	90.455 ± 0.345	90.327 ± 0.652
% LC	0.238 ± 0.001	0.196 ± 0.017	0.227 ± 0.002	0.219 ± 0.011	0.230 ± 0.001	0.218 ± 0.003	0.231 ± 0.003	0.226 ± 0.001	0.224 ± 0.001

**Table 7:** Olive tail moments for formulations F8 and F9 in both cell lines, ARPE-19 and HMC3. Olive tail moment values describe the variations in DNA distribution within the comet tail. Values are presented as the mean ± standard deviation (S.D).

Olive Tail Moment (OTM)		
Formulation	ARPE-19	HMC3
Control	4.77 ± 8.16	3.94 ± 7.45
F8	22.69 ± 27.34	145.45 ± 41.96
F9	17.23 ± 34.83	37.80 ± 41.99

## Data Availability

Not applicable.
